# 3,12-Dimeth­oxy-5,6,9,10-tetra­hydro-[5]helicene-7,8-dicarbo­nitrile

**DOI:** 10.1107/S1600536814014950

**Published:** 2014-07-02

**Authors:** Somboon Sahasithiwat, Thanasat Sooksimuang, Siriporn Kamtonwong, Waraporn Parnchan, Laongdao Kangkaew

**Affiliations:** a National Metal and Materials Technology Center (MTEC), 114 Thailand Science Park, Paholyothin Rd, Klong Luang, Pathumthani 12120, Thailand

**Keywords:** crystal structure

## Abstract

The complete molecule of the title compound, C_26_H_20_N_2_O_2_, is generated by a crystallographic twofold axis. The torsion angle between the terminal and central benzene rings is −32.5 (2)°. The torsion angle along the inner helical rim of the molecule is −18.8 (2)° with each other. The C⋯C distance between the terminal rings is 3.016 (2) Å. In the crystal, weak C—H⋯N hydrogen bonds are observed.

## Related literature   

For the application of a penta­helicene derivative as an emitter in an organic light-emitting diode, see: Sahasithiwat *et al.* (2010 ▶). For related structures, see: McIntosh *et al.* (1954 ▶); Wang *et al.* (1997 ▶); Stammel *et al.* (1999 ▶); Ogawa *et al.* (2003 ▶); Rajapakse *et al.* (2011 ▶). For the synthesis of the title compound, see: Mandal *et al.* (2006 ▶). For general information and applications of helicenes, see: Shen & Chen (2012 ▶); Gingras (2013 ▶).
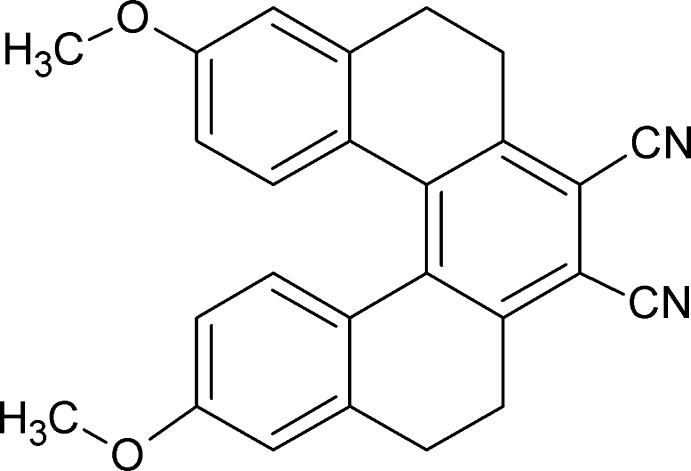


## Experimental   

### 

#### Crystal data  

C_26_H_20_N_2_O_2_
*M* *_r_* = 392.44
Monoclinic, 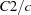

*a* = 17.9533 (7) Å
*b* = 13.5533 (7) Å
*c* = 8.1417 (4) Å
β = 95.785 (2)°
*V* = 1971.00 (16) Å^3^
*Z* = 4
Mo *K*α radiation
μ = 0.08 mm^−1^
*T* = 296 K
0.67 × 0.44 × 0.26 mm

#### Data collection  

Bruker APEXII CCD diffractometer
Absorption correction: multi-scan (*SADABS*; Bruker, 2012 ▶) *T*_min_ = 0.70, *T*_max_ = 0.75
10904 measured reflections
2204 independent reflections
1674 reflections with *I* > 2σ(*I*)
*R*_int_ = 0.025

#### Refinement  

*R*[*F* ^2^ > 2σ(*F* ^2^)] = 0.042
*wR*(*F* ^2^) = 0.137
*S* = 1.07
2204 reflections
136 parameters
H-atom parameters constrained
Δρ_max_ = 0.18 e Å^−3^
Δρ_min_ = −0.19 e Å^−3^

### 

Data collection: *APEX2* (Bruker, 2008 ▶); cell refinement: *SAINT* (Bruker, 2013 ▶); data reduction: *SAINT*; program(s) used to solve structure: *SHELXS2013* (Sheldrick, 2008 ▶); program(s) used to refine structure: *SHELXL2013* (Sheldrick, 2008 ▶); molecular graphics: *ORTEP-3 for Windows* (Farrugia, 2012 ▶) and *Mercury* (Macrae *et al.*, 2006 ▶); software used to prepare material for publication: *WinGX* (Farrugia, 2012 ▶) and *publCIF* (Westrip, 2010 ▶).

## Supplementary Material

Crystal structure: contains datablock(s) I. DOI: 10.1107/S1600536814014950/nr2052sup1.cif

Structure factors: contains datablock(s) I. DOI: 10.1107/S1600536814014950/nr2052Isup2.hkl

Supporting information file. DOI: 10.1107/S1600536814014950/nr2052Isup3.mol

CCDC reference: 1010118

Additional supporting information:  crystallographic information; 3D view; checkCIF report

## Figures and Tables

**Table 1:** Hydrogen-bond geometry (Å, °)

*D*—H⋯*A*	*D*—H	H⋯*A*	*D*⋯*A*	*D*—H⋯*A*
C1—H1⋯N1^i^	0.93	2.79	3.466 (2)	131
C4—H4⋯N1^ii^	0.93	2.86	3.742 (2)	160

Symmetry codes: (i) 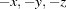; (ii) 
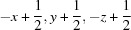
.

**Fig. 1.**
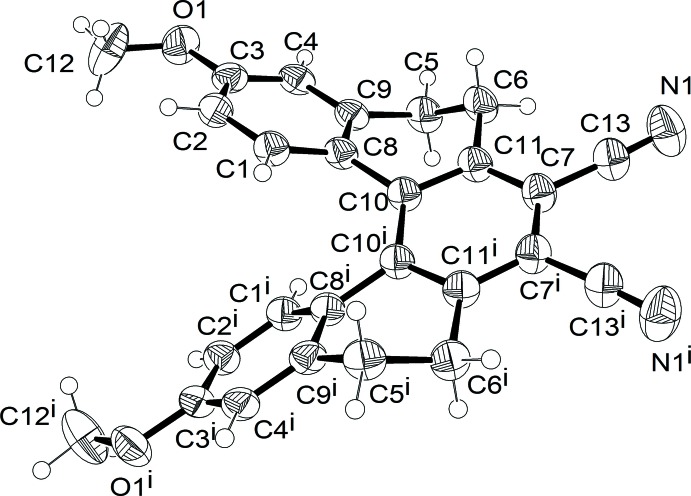
The molecular structure of the title compound showing 50% probability displacement ellipsoids for non-H atoms. Symmetry code: (i) -x,y,1/2 - z.

**Fig. 2.**
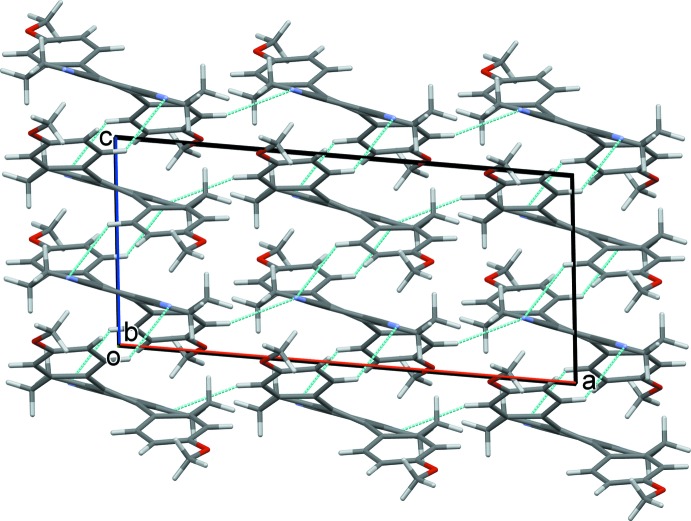
Packing diagram of the title compound projected down the b axis showing hydrogen bonds as dashed lines.

**Fig. 3.**
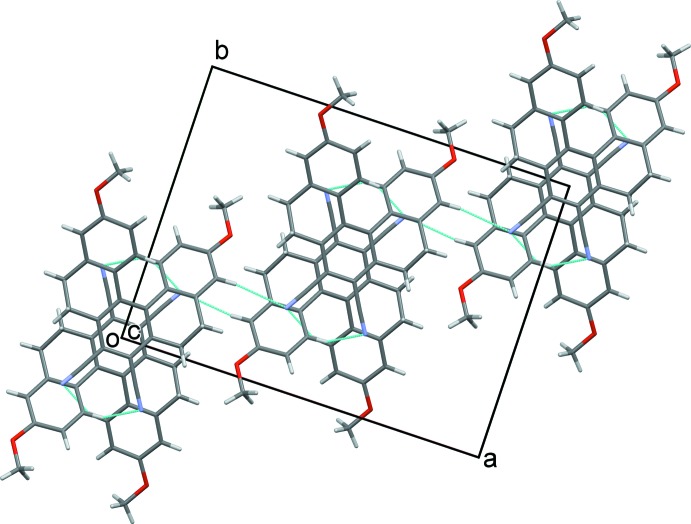
Packing diagram of the title compound projected down the c axis showing hydrogen bonds as dashed lines.

## supplementary crystallographic information

### S1. Comment

Helicenes are polycyclic aromatic hydro­carbons (PAHs) that consist of ortho-fused aromatic rings arranged in helical chiraliry. Applications of helicenes are ranging from catalyst to molecular machines. (Shen *et al.*, 2012; Gingras, 2013). The title compound is a derivative of penta­helicene in which two electron donor and two electron acceptor groups are added into the structure in order to improve its fluorescence quantum yield. Moreover, two rings connected to the central benzene ring are not fully aromatized and in a twist conformation. An application of a similar compound as an emitter for a light-emitting diode was reported (Sahasithiwat *et al.*, 2010).

The geometric parameters of the title molecule agree well with reported similar structures (McIntosh *et al.*, 1954; Wang *et al.*, 1997; Stammel *et al.*, 1999; Rajapakse *et al.*, 2011). Half a molecule of the title compound belongs to asymmetric unit and a molecule is completed by the crystallographic twofold axis as shown in Figure 1. The dihedral angles [C1—C8—C10—C10^i^ and C1^i^—C8^i^—C10^i^—C10] between a terminal and a central benzene ring are -32.5 (2)°. The two non-fully aromatized rings make a dihedral angle [C8—C10—C10^i^—C8^i^] of -18.8 (2)° with each other. The distance of the terminal rings as defined by C1···C1^i^ distance of 3.016 (2)Å is causing by steric repultion of hydrogens on these carbons.

The crystal packing as shown in Figure 2 reveals that the molecules are linked through a network of weak C—H···N [C1—H1···N1(-x,-y,-z)] inter­molecular inter­action yielding racemic columns. Moreover, the other weak C—H···N [C4—H4···N1(-x+1/2,y,-z+1/2)] inter­molecular inter­actions resulted in holding the columns together as exhibited in Figure 3.

### S2. Experimental

A mixture of 6,6'-di­meth­oxy-3,4,3',4'-tetra­hydro­[1,1']binaphthalenyl (9.761 g, 30.7 mmol) and fumaro­nitrile (3.594 g, 46.0 mmol) was stirred and heated at 110 °C under argon atmosphere for 12 h. The resulting mixture was purified by column chromatography (SiO_2_, 7734, 3:2 CH_2_Cl_2_-hexane, 1.8 L) to give a yellow foam of pure Diels-Alder adduct (6.673 g, 55% yield, mp. 139-141 °C). The Diels-Alder product (6.535 g, 16.6 mmol), 2,3-di­chloro-5,6-di­cyano-1,4-benzo­quinone­(8.280 g, 36.5 mmol) and xylene (120 ml) was stirred and refluxed for 6 h. The mixture was cooled to room temperature and filtered off. Filtrate was evaporated to give crude product which was purified by column chromatography (SiO_2_, 7734, toluene, 1.2 L) to give a yellow solid product (3.934 g, 61% yield, mp. 263-264 °C). This compound was characterized by FTIR, ^1^H-NMR, and ^13^C-NMR. Crystals of the title compound suitable for X-ray analysis was obtained by slow evaporation of a chloro­form-hexane solution.

### S2.1. Refinement

All hydrogen atoms were placed in calculated positions and treated as riding atoms with C—H distances of 0.93 Å,0.97 Å, and 0.96 Å for aryl, methyl­ene, and methyl H atoms, respectively. The H atoms were assigned Uiso = 1.2 Ueq(C) for aryl H, Uiso = 1.2 Ueq(C) for methyl­ene H, and Uiso = 1.5 Ueq(C) for methyl H.

### Crystal data

**Table:**

C_26_H_20_N_2_O_2_	*F*(000) = 824
*M**_r_* = 392.44	*D*_x_ = 1.322 Mg m^−^^3^
Monoclinic, *C*2/*c*	Mo *K*α radiation, λ = 0.71073 Å
*a* = 17.9533 (7) Å	Cell parameters from 3787 reflections
*b* = 13.5533 (7) Å	θ = 2.3–27.1°
*c* = 8.1417 (4) Å	µ = 0.08 mm^−^^1^
β = 95.785 (2)°	*T* = 296 K
*V* = 1971.00 (16) Å^3^	Block, green
*Z* = 4	0.67 × 0.44 × 0.26 mm

### Data collection

**Table:**

Bruker APEXII CCD diffractometer	1674 reflections with *I* > 2σ(*I*)
Radiation source: sealed tube	*R*_int_ = 0.025
φ and ω scans	θ_max_ = 27.3°, θ_min_ = 2.3°
Absorption correction: multi-scan (*SADABS*; Bruker, 2012)	*h* = −23→21
*T*_min_ = 0.70, *T*_max_ = 0.75	*k* = −17→17
10904 measured reflections	*l* = −10→10
2204 independent reflections	

### Refinement

**Table:**

Refinement on *F*^2^	0 restraints
Least-squares matrix: full	Hydrogen site location: inferred from neighbouring sites
*R*[*F*^2^ > 2σ(*F*^2^)] = 0.042	H-atom parameters constrained
*wR*(*F*^2^) = 0.137	*w* = 1/[σ^2^(*F*_o_^2^) + (0.0693*P*)^2^ + 0.772*P*] where *P* = (*F*_o_^2^ + 2*F*_c_^2^)/3
*S* = 1.07	(Δ/σ)_max_ < 0.001
2204 reflections	Δρ_max_ = 0.18 e Å^−^^3^
136 parameters	Δρ_min_ = −0.19 e Å^−^^3^

### Special details

**Table:**

Geometry. All e.s.d.'s (except the e.s.d. in the dihedral angle between two l.s. planes) are estimated using the full covariance matrix. The cell e.s.d.'s are taken into account individually in the estimation of e.s.d.'s in distances, angles and torsion angles; correlations between e.s.d.'s in cell parameters are only used when they are defined by crystal symmetry. An approximate (isotropic) treatment of cell e.s.d.'s is used for estimating e.s.d.'s involving l.s. planes.

### Fractional atomic coordinates and isotropic or equivalent isotropic displacement parameters (Å^2^)

**Table:**

	*x*	*y*	*z*	*U*_iso_*/*U*_eq_	
C4	0.18794 (7)	0.30146 (11)	0.13412 (18)	0.0409 (3)	
H4	0.2399	0.3047	0.1479	0.049*	
C10	0.03622 (7)	0.12362 (10)	0.22237 (18)	0.0374 (3)	
C2	0.06956 (8)	0.37374 (11)	0.03575 (19)	0.0419 (4)	
H2	0.0421	0.4247	−0.0174	0.050*	
C8	0.07336 (7)	0.21339 (10)	0.16691 (17)	0.0370 (3)	
O1	0.18785 (6)	0.46083 (8)	0.02479 (16)	0.0572 (3)	
C9	0.15209 (7)	0.21797 (10)	0.18425 (17)	0.0382 (3)	
C1	0.03382 (7)	0.29099 (10)	0.08781 (18)	0.0399 (3)	
H1	−0.0181	0.2870	0.0695	0.048*	
C6	0.15632 (8)	0.03662 (11)	0.1795 (2)	0.0465 (4)	
H6A	0.1598	0.0358	0.0613	0.056*	
H6B	0.1819	−0.0213	0.2269	0.056*	
C7	0.03793 (8)	−0.05492 (10)	0.23454 (19)	0.0416 (4)	
C3	0.14727 (8)	0.38012 (10)	0.06373 (18)	0.0416 (4)	
C11	0.07496 (7)	0.03379 (10)	0.21240 (19)	0.0401 (3)	
C5	0.19390 (7)	0.12912 (11)	0.2542 (2)	0.0465 (4)	
H5A	0.1937	0.1280	0.3733	0.056*	
H5B	0.2455	0.1318	0.2287	0.056*	
C13	0.07734 (8)	−0.14666 (11)	0.2179 (2)	0.0476 (4)	
N1	0.11026 (8)	−0.21725 (10)	0.2016 (2)	0.0659 (5)	
C12	0.14758 (12)	0.54725 (14)	−0.0283 (3)	0.0749 (6)	
H12A	0.1822	0.5981	−0.0518	0.112*	
H12B	0.1184	0.5692	0.0572	0.112*	
H12C	0.1150	0.5328	−0.1262	0.112*	

### Atomic displacement parameters (Å^2^)

**Table:**

	*U*^11^	*U*^22^	*U*^33^	*U*^12^	*U*^13^	*U*^23^
C4	0.0256 (6)	0.0491 (8)	0.0494 (8)	−0.0029 (6)	0.0100 (6)	−0.0070 (6)
C10	0.0260 (6)	0.0394 (7)	0.0466 (8)	−0.0002 (5)	0.0033 (5)	−0.0005 (6)
C2	0.0353 (7)	0.0443 (8)	0.0465 (8)	0.0021 (6)	0.0060 (6)	0.0025 (6)
C8	0.0259 (6)	0.0396 (7)	0.0460 (8)	0.0002 (5)	0.0068 (5)	−0.0018 (6)
O1	0.0440 (6)	0.0520 (7)	0.0778 (8)	−0.0107 (5)	0.0166 (6)	0.0084 (6)
C9	0.0268 (6)	0.0440 (8)	0.0446 (8)	0.0016 (5)	0.0068 (5)	−0.0044 (6)
C1	0.0266 (6)	0.0443 (7)	0.0489 (8)	0.0001 (6)	0.0043 (6)	−0.0007 (6)
C6	0.0294 (7)	0.0446 (8)	0.0664 (10)	0.0074 (6)	0.0086 (7)	−0.0007 (7)
C7	0.0345 (7)	0.0388 (7)	0.0507 (9)	0.0031 (6)	−0.0002 (6)	−0.0011 (6)
C3	0.0370 (7)	0.0448 (8)	0.0447 (8)	−0.0063 (6)	0.0128 (6)	−0.0021 (6)
C11	0.0284 (7)	0.0420 (8)	0.0497 (8)	0.0032 (5)	0.0028 (6)	−0.0007 (6)
C5	0.0251 (6)	0.0520 (9)	0.0623 (10)	0.0043 (6)	0.0043 (6)	0.0005 (7)
C13	0.0370 (8)	0.0427 (8)	0.0618 (10)	0.0005 (6)	−0.0008 (7)	−0.0022 (7)
N1	0.0529 (9)	0.0487 (8)	0.0946 (12)	0.0102 (7)	−0.0004 (8)	−0.0082 (8)
C12	0.0666 (12)	0.0661 (12)	0.0881 (15)	−0.0194 (9)	−0.0103 (10)	0.0366 (11)

### Geometric parameters (Å, º)

**Table:**

C4—C3	1.383 (2)	C1—H1	0.9300
C4—C9	1.3836 (19)	C6—C11	1.5120 (19)
C4—H4	0.9300	C6—C5	1.521 (2)
C10—C11	1.4085 (18)	C6—H6A	0.9700
C10—C10^i^	1.418 (3)	C6—H6B	0.9700
C10—C8	1.4796 (19)	C7—C11	1.3943 (19)
C2—C1	1.3798 (19)	C7—C7^i^	1.410 (3)
C2—C3	1.393 (2)	C7—C13	1.444 (2)
C2—H2	0.9300	C5—H5A	0.9700
C8—C1	1.3903 (19)	C5—H5B	0.9700
C8—C9	1.4076 (18)	C13—N1	1.1392 (19)
O1—C3	1.3692 (17)	C12—H12A	0.9600
O1—C12	1.421 (2)	C12—H12B	0.9600
C9—C5	1.5005 (19)	C12—H12C	0.9600
			
C3—C4—C9	120.71 (12)	H6A—C6—H6B	108.1
C3—C4—H4	119.6	C11—C7—C7^i^	120.32 (8)
C9—C4—H4	119.6	C11—C7—C13	119.08 (13)
C11—C10—C10^i^	119.48 (8)	C7^i^—C7—C13	120.54 (8)
C11—C10—C8	116.93 (12)	O1—C3—C4	116.15 (12)
C10^i^—C10—C8	123.54 (7)	O1—C3—C2	123.99 (14)
C1—C2—C3	119.29 (13)	C4—C3—C2	119.85 (13)
C1—C2—H2	120.4	C7—C11—C10	119.56 (12)
C3—C2—H2	120.4	C7—C11—C6	121.77 (12)
C1—C8—C9	118.28 (12)	C10—C11—C6	118.67 (12)
C1—C8—C10	122.59 (12)	C9—C5—C6	108.96 (12)
C9—C8—C10	118.98 (12)	C9—C5—H5A	109.9
C3—O1—C12	117.57 (12)	C6—C5—H5A	109.9
C4—C9—C8	119.92 (13)	C9—C5—H5B	109.9
C4—C9—C5	122.58 (12)	C6—C5—H5B	109.9
C8—C9—C5	117.49 (12)	H5A—C5—H5B	108.3
C2—C1—C8	121.71 (13)	N1—C13—C7	177.50 (18)
C2—C1—H1	119.1	O1—C12—H12A	109.5
C8—C1—H1	119.1	O1—C12—H12B	109.5
C11—C6—C5	110.32 (12)	H12A—C12—H12B	109.5
C11—C6—H6A	109.6	O1—C12—H12C	109.5
C5—C6—H6A	109.6	H12A—C12—H12C	109.5
C11—C6—H6B	109.6	H12B—C12—H12C	109.5
C5—C6—H6B	109.6		
			
C11—C10—C8—C1	145.00 (15)	C9—C4—C3—C2	−3.0 (2)
C10^i^—C10—C8—C1	−32.5 (3)	C1—C2—C3—O1	−176.54 (14)
C11—C10—C8—C9	−30.49 (19)	C1—C2—C3—C4	3.8 (2)
C10^i^—C10—C8—C9	152.05 (18)	C7^i^—C7—C11—C10	0.5 (3)
C3—C4—C9—C8	−1.3 (2)	C13—C7—C11—C10	177.68 (15)
C3—C4—C9—C5	177.21 (14)	C7^i^—C7—C11—C6	179.84 (17)
C1—C8—C9—C4	4.7 (2)	C13—C7—C11—C6	−3.0 (2)
C10—C8—C9—C4	−179.60 (13)	C10^i^—C10—C11—C7	8.9 (3)
C1—C8—C9—C5	−173.89 (13)	C8—C10—C11—C7	−168.68 (14)
C10—C8—C9—C5	1.79 (19)	C10^i^—C10—C11—C6	−170.49 (16)
C3—C2—C1—C8	−0.3 (2)	C8—C10—C11—C6	11.9 (2)
C9—C8—C1—C2	−3.9 (2)	C5—C6—C11—C7	−147.72 (15)
C10—C8—C1—C2	−179.43 (13)	C5—C6—C11—C10	31.6 (2)
C12—O1—C3—C4	−172.31 (16)	C4—C9—C5—C6	−136.89 (14)
C12—O1—C3—C2	8.0 (2)	C8—C9—C5—C6	41.68 (18)
C9—C4—C3—O1	177.34 (13)	C11—C6—C5—C9	−57.20 (17)

Symmetry code: (i) −*x*, *y*, −*z*+1/2.

### Hydrogen-bond geometry (Å, º)

**Table:**

*D*—H···*A*	*D*—H	H···*A*	*D*···*A*	*D*—H···*A*
C1—H1···N1^ii^	0.93	2.79	3.466 (2)	131
C4—H4···N1^iii^	0.93	2.86	3.742 (2)	160

Symmetry codes: (ii) −*x*, −*y*, −*z*; (iii) −*x*+1/2, *y*+1/2, −*z*+1/2.
